# Impact of early diagnosis on surgical outcomes in patients with Loeys-Dietz syndrome

**DOI:** 10.3389/fcvm.2024.1429222

**Published:** 2024-08-16

**Authors:** Hongsun Kim, Jun Ho Lee, Su Ryeun Chung, Pyo Won Park, Taek Kyu Park, I-Seok Kang, June Huh, Duk-Kyung Kim, Yang Hyun Cho, Kiick Sung

**Affiliations:** ^1^ Department of Cardiovascular Surgery, Mayo Clinic, Rochester, MN, United States; ^2^ Department of Thoracic and Cardiovascular Surgery, Korea University Anam Hospital, Korea University College of Medicine, Seoul, Republic of Korea; ^3^ Department of Thoracic and Cardiovascular Surgery, Samsung Medical Center, Sungkyunkwan University School of Medicine, Seoul, Republic of Korea; ^4^ Department of Thoracic and Cardiovascular Surgery, Incheon Sejong Hospital, Incheon, Republic of Korea; ^5^ Division of Cardiology, Department of Medicine, Samsung Medical Center, Sungkyunkwan University School of Medicine, Seoul, Republic of Korea; ^6^ Department of Pediatrics, Samsung Medical Center, Sungkyunkwan University School of Medicine, Seoul, Republic of Korea; ^7^ Division of Cardiology, Department of Medicine, Samsung Changwon Hospital, Sungkyunkwan University School of Medicine, Seoul, Republic of Korea

**Keywords:** aortic aneurysm, aortic dissection, connective tissue disease, Loeys-Dietz syndrome, early diagnosis

## Abstract

**Background:**

This study aimed to investigate the influence of early diagnosis (ED) on surgical outcomes in patients definitively diagnosed with Loeys-Dietz syndrome (LDS).

**Methods:**

A retrospective review was conducted on 38 patients with LDS who underwent aortic surgery at our institution between January 1995 and June 2022. The primary endpoint was freedom from aortic reoperation.

**Results:**

Among the patients, the median age at the initial surgery was 33 (range: 39–44) years, and 23 (60.5%) patients were male. Twenty-one (55.3%; aortic dissection or rupture (*n* = 2) and aneurysm (*n* = 19)) patients were diagnosed with LDS before the initial surgery (ED group). Meanwhile, the remaining 17 (44.7%; aortic dissection or rupture (*n* = 13) and aneurysm (*n* = 4)) patients were after surgery [delayed diagnosis (DD) group]. The ED group had significantly lower rates of emergency surgery and concomitant arch procedure (*P* < .001, respectively) but a higher rate of valve-sparing root surgery (*P* = .018) compared to the DD group. No in-hospital mortality was observed in either group. Nevertheless, the ED group had a shorter postoperative hospital stay (median difference: 3 days, *P* = .032) and a lower rate of aortic reoperation (*P* = .013).

**Conclusion:**

Early detection of LDS may help in preventing acute aortic syndrome, reducing the risk of aortic reoperation, and potentially shortening hospital stay. Careful medical management before surgery could contribute to better clinical outcomes and an improved quality of life for patients with LDS.

## 1 Introduction

Loeys-Dietz syndrome (LDS) is a rare genetic disorder that can have devastating consequences if left untreated. LDS was first reported in 2005 (1, 2). This syndrome is associated with a high risk of aortic aneurysms and dissections, thereby leading to significant morbidity and mortality. Individuals with a family history of LDS are recommended for familial genetic screening (3–5). Nonetheless, several patients with LDS initially present with acute aortic syndrome, including aortic dissection or rupture.

However, there is a paucity of research on the association between the timing of diagnosis and surgical outcomes in patients with LDS. We hypothesized that early LDS diagnosis could avoid surgery for acute aortic syndrome and result in better surgical outcomes. Therefore, we reviewed our 27-year surgical experience with LDS.

## 2 Materials and methods

### 2.1 Study population

**Figure 1:**
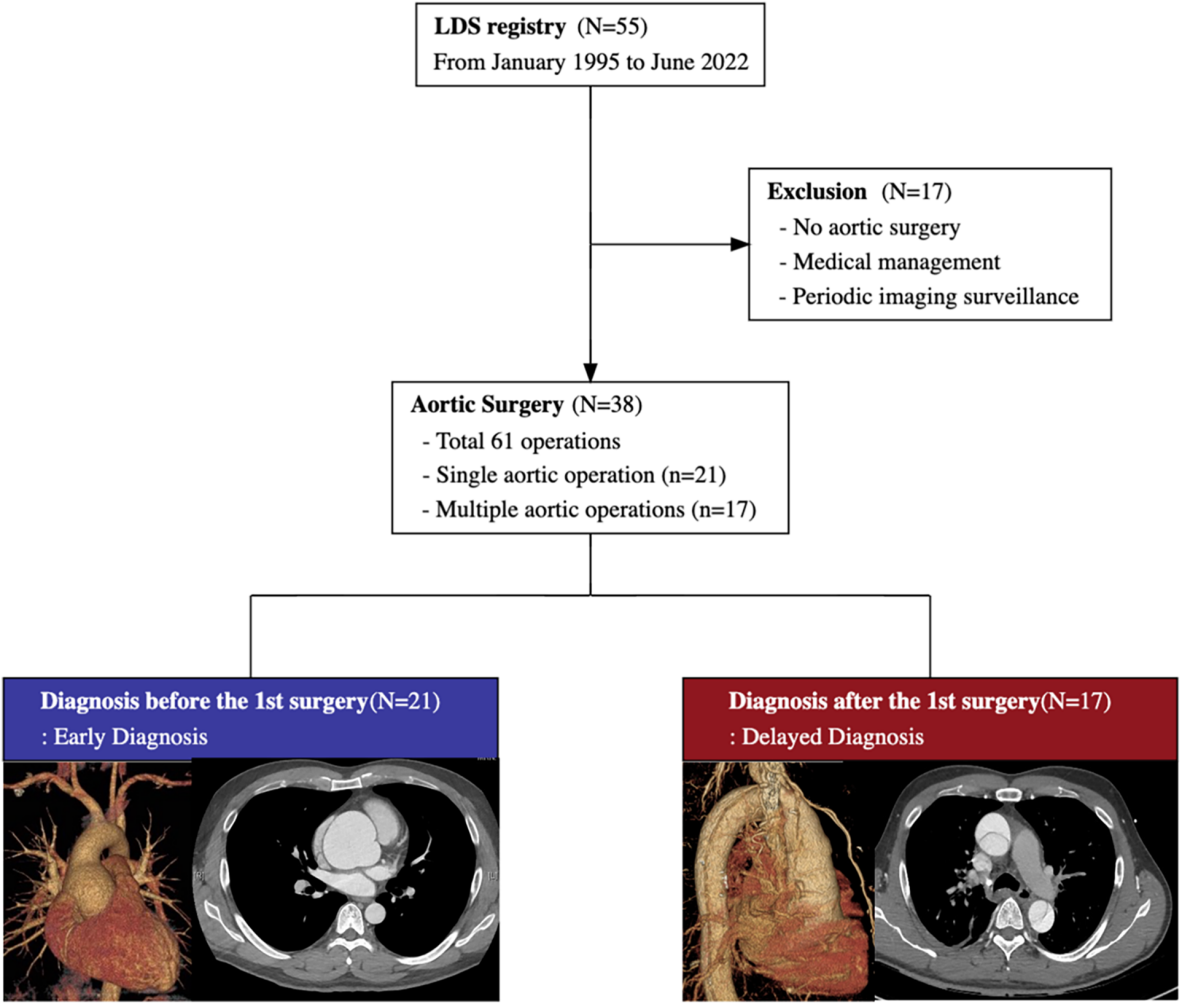
The study flow diagram summarizes the medical and surgical treatment of 55 patients with Loeys-Dietz syndrome. Among 38 patients with aortic surgery, 21 patients are diagnosed before the initial aortic surgery, and the remaining 17 are diagnosed after the initial surgery.

We conducted a retrospective review of consecutive patients diagnosed with LDS at Samsung Medical Center in South Korea between January 1995 and June 2022. Fifty-five patients with LDS diagnosis were noted, of whom thirty-eight underwent aortic surgery (Figure 1). Among them, 21 (55.3%) patients were genetically diagnosed with LDS before their initial surgery [early diagnosis (ED) group], whereas the remaining 17 (44.7%) patients were not [delayed diagnosis (DD) group].

### 2.2 Data collection

During diagnosis and the perioperative period, we documented relevant clinical data including age, sex, height, weight, family history of LDS and aortic complication, genetic mutation type, history of hypertension, preoperative echocardiographic findings, and operative variables. LDS types were classified according to the gene mutation type identified as follows: LDS1, LDS2, LDS3, LDS4, and LDS5 for transforming growth factor beta receptor 1 (*TGFBR1)*, *TGFBR2*, *SMAD3*, transforming growth factor beta 2 (*TGFB2*), and *TGFB3.* Furthermore, data on surgical outcomes, including operative mortality, reoperation for bleeding, reintubation, atrial fibrillation, stroke, mechanical ventilation duration, length of intensive care unit stay, and length of hospital stay, were recorded. Operative mortality was defined as any death, regardless of cause, occurring within 30 days postoperatively in or out of the hospital, and after 30 days during the same hospitalization after the surgery.

### 2.3 Follow-up data and outcomes

Follow-up data were obtained via examination during clinic visits, review of medical records, and imaging. Data on mortality in patients who were lost to follow-up were confirmed on the basis of the National Health Insurance Data. The primary endpoint was freedom from aortic reoperation. The secondary endpoints included postoperative in-hospital morbidity and mortality, as well as overall mortality.

### 2.4 Operative indications and techniques

In patients with Stanford type A acute aortic syndrome and those with signs of aortic rupture, emergent aortic surgery was performed according to the treatment guidelines and consensus (3–6). In cases of aortic aneurysms, the guidelines recommend a low threshold for performing aortic surgery in patients with hereditary aortic disease, including LDS, compared with those without (3–5). At our institution, prophylactic aortic surgery was considered at different aortic diameter thresholds on the basis of LDS type. In cases involving an aortic root diameter of 40 mm in patients with LDS1 and LDS2, 40–42 mm in those with LDS3, and 42–45 mm in those with LDS4 and LDS5, prophylactic surgery was considered. Moreover, prophylactic surgery was performed in cases involving an ascending aorta with a diameter of 42–50 mm and an arch and descending aorta with a diameter of 55–60 mm.

In cases of acute Stanford type A aortic dissection, right axillary artery cannulation was routinely performed, followed by standard median sternotomy. Previous reports have described the surgical approach for acute type A aortic dissection in detail (7, 8). Patients undergo surgical procedures via median sternotomy for aortic root replacement. The choice between a modified Bentall procedure and valve-sparing root replacement is based on the quality of the aortic valve leaflets and the surgeon's technical experience. The David reimplantation technique for valve-sparing replacement was preferred in patients with LDS owing to aortic annulus stabilization (7, 9). A left posterior thoracotomy is performed in descending thoracic aorta repair, and the thoracotomy incision is extended and connected to a median laparotomy in thoracoabdominal aorta repair (10). Cerebrospinal fluid drainage and motor-evoked potential monitoring are routinely performed for thoracoabdominal aorta repair, but only in patients at high risk of spinal injury in descending thoracic aorta repair.

### 2.5 Statistical analyses

Continuous variables were presented as medians with interquartile ranges (IQRs) and categorical variables were presented as numbers with percentages. The Mann–Whitney U test was used to compare continuous variables, and the chi-squared test or Fisher's exact test was utilized to analyze categorical data. The Kaplan–Meier method was used to estimate freedom from reoperation, and the log-rank test was applied to compare outcomes between patients with ED and those with DD. All tests were two-tailed, and *P*-values of <0.05 were considered statistically significant. The R software version 4.2.1 (R Foundation for Statistical Computing, Vienna, Austria) was used for all analyses.

## 3 Results

### 3.1 Demographic characteristics

In this study, 38 patients were enrolled, and their baseline characteristics are presented in Table 1. The median age of the patients was 33 (IQR, 19–41) years, and 23 (60.5%) patients were male. The *TGFBR2* mutation (LDS2, 50%) was the most common type of mutation.

**Table 1:** Baseline characteristics of patients who underwent the initial aortic surgery.

Variables	Total patients (*n* = 38)	Early diagnosis (*n* = 21)	Delayed diagnosis (*n* = 17)	*P* value
Age	33 (19.0–41.0)	34.4 (17.2–39.6)	31.9 (21.6–45.6)	.601
Male	23 (60.5)	15 (71.4)	8 (47.1)	.126
Height (cm, *n* = 29)	174.0 (165.0–180.0)	175.0 (166.3–180.9)	173.5 (166.2–174.8)	.613
Weight (kg, *n* = 29)	69.0 (55.0–78.0)	65.2 (53.9–79.7)	69.5 (57.0–74.9)	.982
BMI (kg/m^2^, *n* = 29)	22.2 (20.2–24.8)	22.0 (18.6–25.0)	23.1 (21.5–24.7)	.330
Family history				
LDS	17 (44.7)	10 (47.6)	7 (41.2)	.691
Aortic dissection/rupture	13 (34.2)	8 (38.1)	5 (29.4)	.575
SCD	13 (34.2)	6 (28.6)	7 (41.2)	.415
Gene				>.999
TGFBR1 (LDS type 1)	11 (28.9)	6 (28.6)	5 (29.4)	
TGFBR2 (LDS type 2)	19 (50.0)	10 (47.6)	9 (52.9)	
SMAD3 (LDS type 3)	5 (13.2)	3 (14.3)	2 (11.8)	
TGFB2 (LDS type 4)	2 (5.3)	1 (4.8)	1 (5.9)	
TGFB3 (LDS type 5)	1 (2.6)	1 (4.8)	0 (0.0)	
Hypertension	11 (28.9)	7 (33.3)	4 (23.5)	.721
LVEF (%, *n* = 31)	65.0 (60.5–68.0)	65.0 (61.5–67.0)	65.0 (60.5–68.0)	.804
SOV diameter (mm, *n* = 29)	46.5 (41.3–54.0)	45.5 (40.7–51.9)	52.7 (46.1–62.5)	.113
AR ≥mild (*n* = 33)	14 (42.4)	5 (23.8)	9 (75.0)	.004

Data are presented as medians (IQRs) or as *n* (%). BMI, body mass index; LDS, Loeys-Dietz syndrome; LVEF, left ventricular ejection fraction; SCD, sudden cardiac death; SOV, sinus of Valsalva, AR; aortic regurgitation.

We classified the patients into the ED and DD groups according to the timing of LDS diagnosis and the initial aortic surgery (Table 1). The ED group was composed of 21 (55.3%) patients genetically diagnosed with LDS before their initial aortic surgery, and the median duration from diagnosis to surgery was 3.1 (IQR, 1.9–11.0) months. In the DD group, 17 (44.7%) patients underwent aortic surgery before the diagnosis. In our cohort, 15 (39.5%) patients were female. In the ED group, 28.6% (6/21) were female, and in the DD group, 52.9% (9/17) were female. The aortic diameter at the time of initial aortic surgery for the entire cohort was 46.5 mm (IQR, 41.3–54.0), and the difference in diameter between genders was not statistically significant (female: 46.1 mm [IQR, 41.5–50.6]; male: 47.0 mm [IQR, 41.2–59.4]; *P* = .696). The baseline demographic characteristics, except for preoperative aortic insufficiency, which was more commonly observed in the DD group, were similar between the two groups.

### 3.2 Findings of the aortic surgery

A total of 61 aortic surgeries were performed on 38 patients enrolled. Twenty-one patients underwent single aortic surgery, and the other 17 patients underwent multiple aortic surgeries. The highest number of surgeries performed on a single patient was four. The findings at the initial aortic surgery are shown in Table 2. Moreover, 2 (9.5%) patients in the ED group and 13 (76.5%) in the DD group underwent emergent surgery for aortic dissection or rupture (*P* < .001). The proportion of patients who underwent aortic root surgery was comparable between the two groups (85.7% vs. 64.7%, *P* = .249). However, among these 29 root surgery cases, more valve-sparing root surgery was performed in the ED group (77.8% vs. 27.3%, *P* = .018). Only one (4.8%) case of arch surgery and associated circulatory arrest was noted in the ED group, however, in the DD group, 11 (64.7%) patients underwent arch surgery and circulatory arrest (*P* < .001). No significant differences were observed between the two groups regarding the rate of descending aortic surgery and concomitant procedure, cardiopulmonary bypass time, cross-clamp time, and body temperature.

**Table 2:** Operative findings of patients at the initial aortic surgery.

Variables	Total patients (*n* = 38)	Early diagnosis (*n* = 21)	Delayed diagnosis (*n* = 17)	*P* value
Aortic diagnosis				<.001
Acute dissection/rupture	15 (39.5)	2 (9.5)	13 (76.5)	
Aneurysm	23 (60.5)	19 (90.5)	4 (23.5)	
Emergency	15 (39.5)	2 (9.5)	13 (76.5)	<.001
Operative procedure				
Root	29 (76.3)	18 (85.7)	11 (64.7)	.249
Bentall	12 (31.6)	4 (19.0)	8 (47.1)	
David	17 (44.7)	14 (66.7)	3 (17.6)	
Ascending aorta	34 (89.5)	19 (90.5)	15 (88.2)	>.999
Arch	12 (31.6)	1 (4.8)	11 (64.7)	<.001
Hemiarch	6 (15.8)	0 (0.0)	6 (35.3)	
Partial arch	3 (7.9)	1 (4.8)	2 (11.8)	
Total arch	3 (7.9)	0 (0.0)	3 (17.6)	
Descending	4 (10.5)	2 (9.5)	2 (11.8)	.640
Descending thoracic aorta	2 (5.3)	0 (0.0)	2 (11.8)	
TAA	1 (2.6)	1 (4.8)	0 (0.0)	
AAA	1 (2.6)	1 (4.8)	0 (0.0)	
Concomitant procedure	10 (26.3)	6 (28.6)	4 (23.5)	>.999
CPB				
CPB (minutes, *n* = 30)	178.0 (156.0–211.0)	163.0 (152.5–210.5)	197.0 (178.0–208.5)	.272
ACC (minutes, *n* = 30)	144.0 (126.0–172.0)	140.0 (124.0–169.0)	156.0 (128.5–173.0)	.667
Circulatory arrest	12 (31.6)	1 (4.8)	11 (64.7)	<.001
Rectal temperature (℃, *n* = 30)	28.5 (25.8–30.4)	29.0 (27.0–30.9)	25.6 (22.6–29.9)	.102

Data are presented as medians (IQRs), or as *n* (%). AAA, abdominal aortic aneurysm; ACC, aortic cross clamp; ASD, atrial septal defect; AVP, aortic vavuloplasty; CABG, coronary artery bypass graft; CPB, cardiopulmonary bypass; MV surgery, mitral valve surgery; TAAA, thoracoabdominal aortic aneurysm; TEVAR, thoracic endovascular aortic repair.

### 3.3 Operative outcomes and follow-up

The operative outcomes following initial surgeries are shown in Table 3. There was no operative mortality, and only one patient who had a previous history of stroke and underwent emergent ascending aorta and partial arch replacement suffered a postoperative stroke. No significant differences in terms of the rate of reoperation for bleeding, reintubation, postoperative atrial fibrillation, stroke, and mechanical ventilation duration were noted between the ED and DD groups. However, the ED group had a trend toward a shorter length of intensive care unit stay (22 [IQR, 20–38] vs. 41 [IQR, 26–60] hours, *P* = .106) and experienced a significantly shorter length of hospital stay (8.0 [IQR, 7.0–10.0] vs. 11.0 [IQR, 9.0–13.5] days, *P* = .032) than the DD group.

**Table 3:** Operative outcomes after the initial surgery.

Variables	Total patients (*n* = 38)	Early diagnosis (*n* = 21)	Delayed diagnosis (*n* = 17)	*P* value
Operative mortality	0	0	0	
Reoperation for bleeding (*n* = 31)	0	0	0	
Reintubation (*n* = 31)	1 (3.2)	1 (5.0)	0 (0.0)	>.999
Atrial fibrillation (*n* = 31)	4 (12.9)	3 (15.0)	1 (9.1)	>.999
Stroke (*n* = 36)	1 (2.9)	0 (0.0)	1 (6.7)	.417
MV duration (hours, *n* = 30)	10 (4–16)	10 (4–15)	10 (6–20)	.171
ICU LOS (hours, *n* = 31)	23 (20–47)	22 (20–38)	41 (26–60)	.106
Hospital LOS (days, *n* = 31)	9.0 (7.0–12.0)	8.0 (7.0–10.0)	11.0 (9.0–13.5)	.032
Reoperation event at 5 years	10 (37.8^a^)	1 (14.3^a^)	9 (59.2^a^)	.017
Ascending	3/10 (30.0)	1/1 (100.0)	2/9 (22.2)	.300
Root	2/10 (20.0)	0 (0)	2/9 (22.2)	>.999
Arch	3/10 (30.0)	1/1 (100.0)	2/9 (22.2)	.300
Descending	6/10 (60.0)	0 (0)	6 (66.7)	.400

Data are presented as medians (IQRs), or as *n* (%). ICU, intensive care unit; LOS, length of stay; MV duration, mechanical ventilation duration.

^a^ 5-year reoperation rate calculated using the Kaplan-Meier estimate.

**Figure 2:**
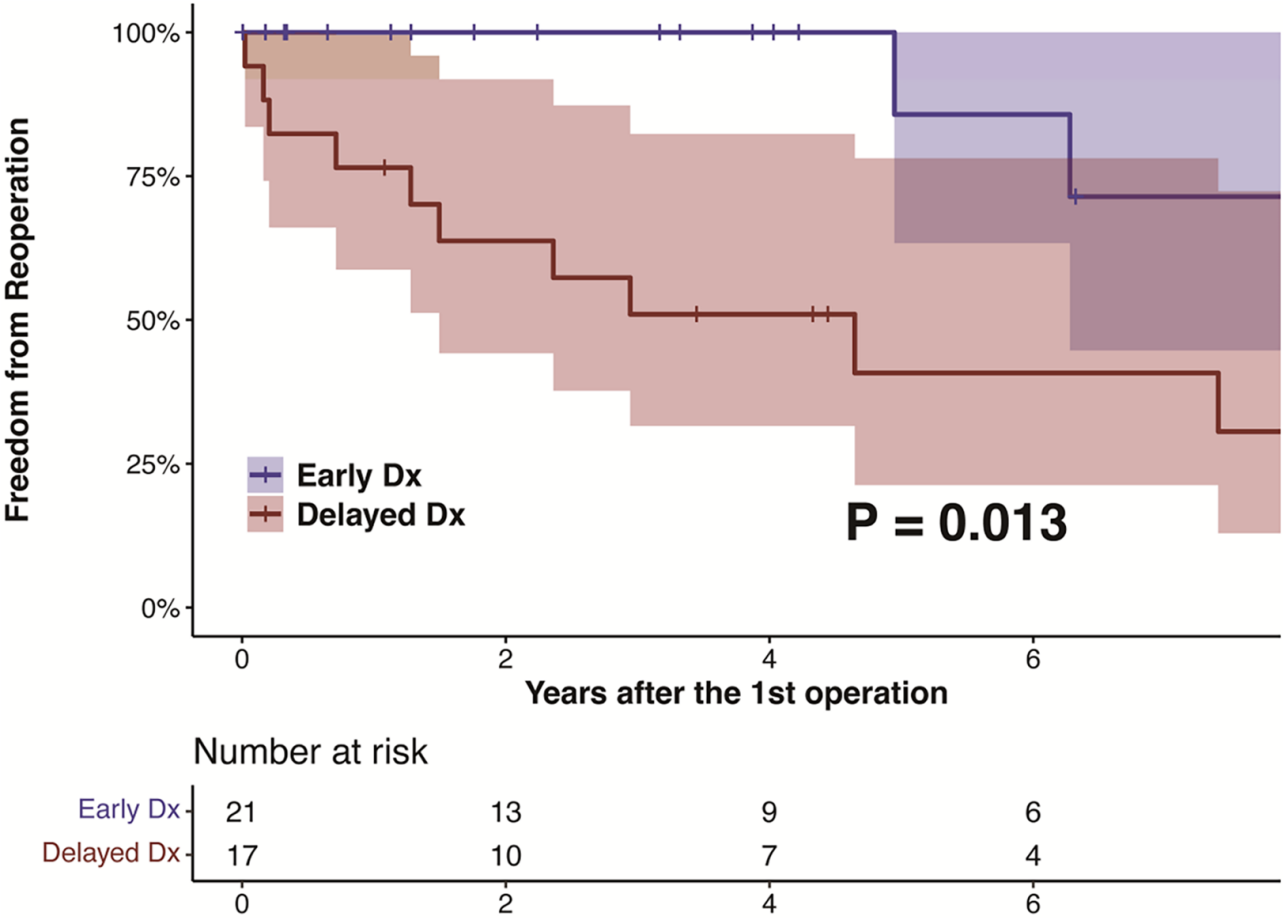
The kaplan-meier estimate and log-rank test for freedom from reoperation in patients with LDS according to the diagnosis before and after their initial aortic surgeries. LDS, Loeys-Dietz syndrome.

**Figure 3:**
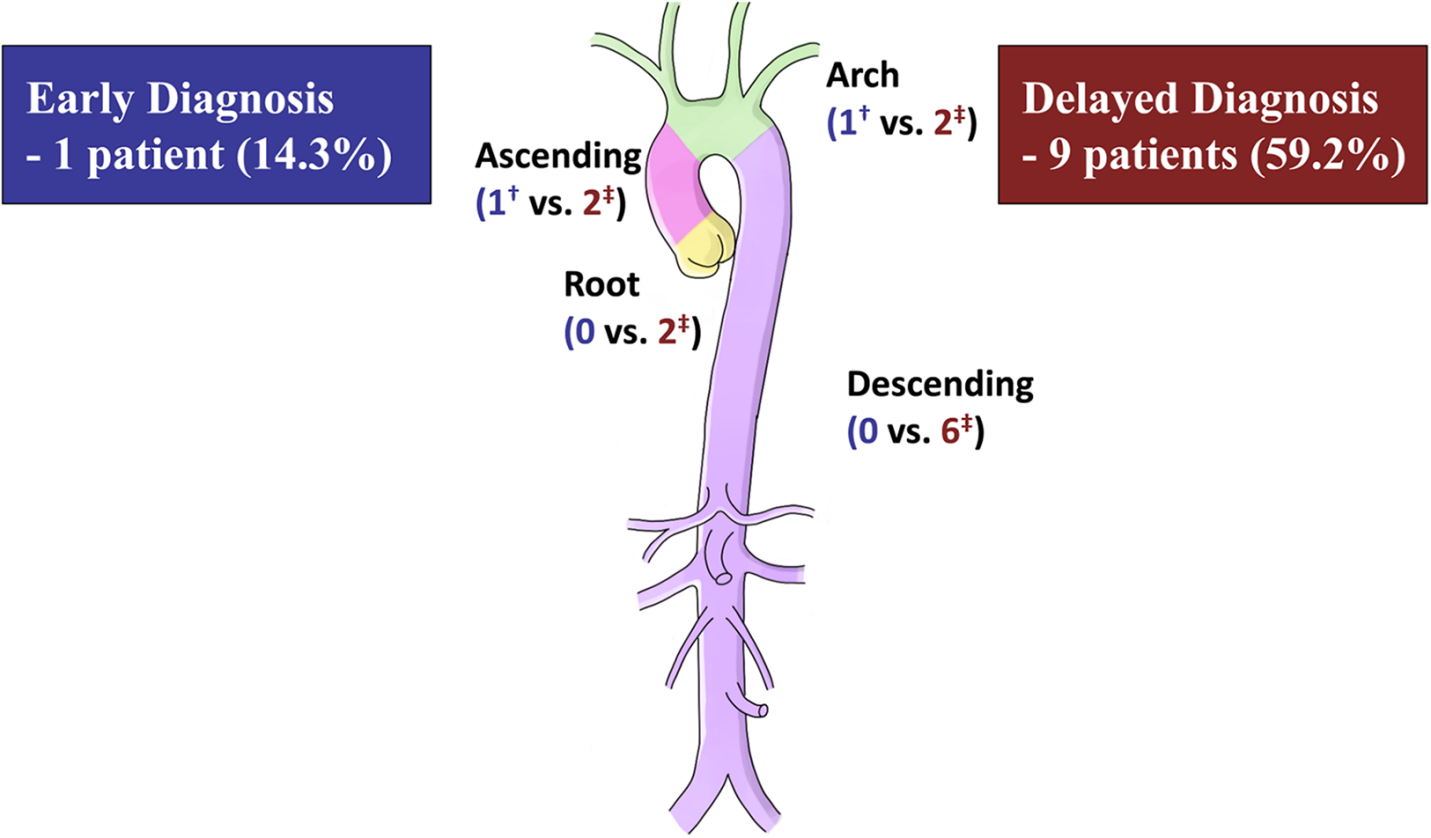
Number of reoperations at 5 years after the initial aortic surgery. ^†^One patient in the early diagnosis group underwent reoperation on the ascending and aortic arch. ^‡^Nine patients in the delayed diagnosis group (including two on the aortic root, two on the ascending aorta, two on the aortic arch, and six on the descending aorta) underwent aortic reoperation.

After discharge, patients were followed up at a median of 50.6 (IQR, 21.1–153.3) months, during which no deaths were recorded. The ED group had a significantly higher rate of freedom from reoperation than the DD group (*P* = .013), as shown in Figure 2. The estimated rates of aortic reoperation at 5 years were 14.3%±13.2% and 59.2%±13.5% in the ED and DD groups, respectively, as shown in Figure 3. One patient in the ED group underwent reoperation on the ascending and aortic arch. And, nine patients in the DD group (including two on the aortic root, two on the ascending aorta, two on the aortic arch, and six on the descending aorta) underwent aortic reoperation.

During the follow-up period, there were 13 reoperations for aneurysm (three in the ED group and ten in the DD group) and four reoperations for dissection (one in the ED group and three in the DD group).

## 4 Discussion

The exact prevalence of LDS is currently unknown and requires further research (1, 2). To identify those at risk, the treatment guidelines recommend genetic surveillance for the first-degree relatives of a confirmed patient (3–5). We compared the outcomes of aortic surgery in patients with LDS diagnosed before or after the surgery. Results showed that early LDS diagnosis before aortic surgery leads to better outcomes, including the reduced rate of emergency surgeries, arch procedures, and shorter length of hospital and stay later aortic reoperation rates. Furthermore, the study showed that early diagnosis may help preserve the patient's aortic valve and prevent the need for life-long anticoagulation, particularly in younger patients.

Our institution strongly recommended genetic testing for patients exhibiting syndromic features of LDS, regardless of prior surgeries. Over a decade ago, Sanger sequencing was used to detect variants in the FBN1, TGFBR1, and TGFBR2 genes. Recently, we have adopted a multigene panel test that includes all genes suspected of causing hereditary aortopathy. Mutations in FBN1, TGFBR1, TGFBR2, SMAD3, and TGFB2 have been identified in approximately 6% to 8% of patients without significant features (5). Our institution performed genetic testing in patients with thoracic aortic diseases who have high-risk features, including age less than 50 years, a family history of thoracic aortic diseases, arterial tortuosity, or a pathologic report of cystic medial degeneration.

The previous guideline recommended aortic surgery at 42 mm by transesophageal echocardiography (internal diameter) or 44–46 mm by computed tomography (external diameter) for TGFBR1/TGFBR2 (LDS 1 or 2) (3). The recent guideline suggested aortic surgery at 45 mm for low-risk LDS 1 or 2, and at 40 mm for high-risk cases, known to be aggressive (5). For LDS 3 (SMAD3), aortic surgery is recommended at 45 mm, while for the relatively mild LDS 4 or 5 (TGFB2 or 3), surgery is recommended at 45 mm and 50 mm, respectively (5). Another previous guideline does not differentiate based on LDS type and recommends aortic surgery for LDS at 42 mm or more (4). Our institution follows surgical indications based on smaller diameters than existing guidelines. This approach is due to our belief that aggressive early surgery improves prognosis and acknowledges that East Asians generally have a smaller body type compared to Europeans or Americans. Therefore, caution is necessary when applying size criteria designed for European or American populations.

The results of this study suggest that the outcomes were primarily influenced by aortic dissection occurrence. However, of note, in this study, the presence of ED and aortic dissection occurrence did not frequently align. Within our cohort, two patients in the ED group, who had not undergone prophylactic surgery, experienced acute aortic dissection and required emergency aortic surgery. In contrast, four patients in the DD group underwent elective aortic surgery for an aneurysm. Further details of these patients are presented in Supplementary Table S1. Among the patients in the ED group, one underwent aortic surgery 10 years following diagnosis, whereas another patient had the surgery 11 months following diagnosis. Both patients experienced favorable outcomes, with no need for reoperation or overall mortality. It is possible that meticulous medical treatments before surgery, including the use of beta blockers and renin-angiotensin-system blockers, played a significant role in achieving these positive results.

A study conducted by Aftab et al. reported that 33 of 53 patients with LDS underwent aortic surgery (11). In patients who initially presented with aortic dissection, the risk of reoperation was high. Moreover, the study showed that the patients with dissection commonly presented with types 1 and 2 LDS, which are the aggressive types (1, 2, 12). However, they did not review the timing of the LDS diagnosis and its impact on clinical outcomes. In our study, 38 of 55 patients with LDS required aortic surgery. Among them, 17 patients underwent multiple aortic surgeries. Patients who underwent surgery frequently presented with types 1 and 2 LDS, and only one patient with type 4 LDS underwent surgical repair prophylactically. The DD group had a higher number of patients with acute dissection and a higher reoperation rate than the ED group, which was consistent with a previous study.

Several studies have recommended aggressive arch procedures for patients with LDS (13, 14). In a recent comparative study, patients with LDS had higher rates of reoperation, particularly for arch reoperation, than those with Marfan syndrome (MFS) (13). The study showed that the cumulative incidence rate of arch reoperation in patients with LDS who underwent their initial surgery without the arch procedure was approximately 60%–70%. Furthermore, another study reported that 20 patients with LDS who underwent elective aortic root procedures did not require aortic arch reoperation (11). However, based on our findings, only one (4.8%) arch surgery was performed on patients with LDS who were diagnosed early before their initial surgery. In our study, the 5-year reoperation rate was 14.3% in patients with ED, which was lower than that (42.8%) reported in the comparative study (13). Therefore, we believe that ED, medical management, and timely surgery can reduce the risk of arch reoperation.

Schoenhoff and colleagues focused on the management of the aortic arch in patients with LDS and MFS (15). Its results showed that patients with nondissected LDS had a higher arch intervention rate than those with nondissected MFS. However, the authors concluded the complete removal of the distal ascending aorta, not beyond the arch, at the initial surgery. Furthermore, we also prefer to replace the ascending aorta as distal as possible in the cases of elective aortic root surgery.

### 4.1 Limitations

This study had several limitations that must be acknowledged. First, this retrospective, non-randomized study was conducted at a single center, which may have led to potential bias and limited the generalizability of our findings. Moreover, surgeons’ various levels of experience and confidence in performing valve-sparing root replacement (VSRR) may have contributed to differences in emergency VSRR between the ED and DD groups. Furthermore, surgeon's preference for the surgical extent could have affected the risk of aortic reoperation. Second, because LDS is a rare condition and has only been recently discovered, the sample size and follow-up period were limited, which could have resulted in underpowered analyses. Further studies involving a large multicenter approach with long-term follow-up may be warranted. Finally, as patients who underwent surgery at other hospitals were included owing to the small sample size limitation, complete data collection was not performed.

## 5 Conclusion

Early detection of LDS may allow for careful medical management and preventive aortic surgery, helping to avoid aortic emergencies and improve the overall clinical course while possibly reducing the need for further surgical interventions. Given the rarity of this disorder, active screening and a high level of suspicion are important for the timely identification of the condition, which might prevent catastrophic aortic events.

## Data Availability

The original contributions presented in the study are included in the article/Supplementary Material, further inquiries can be directed to the corresponding authors.

## References

[1] Loeys BL Chen J Neptune ER Judge DP Podowski M Holm T A syndrome of altered cardiovascular, craniofacial, neurocognitive and skeletal development caused by mutations in TGFBR1 or TGFBR2. Nat Genet. (2005) 37:275–81. 10.1038/ng1511 15731757

[2] Loeys BL Schwarze U Holm T Callewaert BL Thomas GH Pannu H Aneurysm syndromes caused by mutations in the TGF-beta receptor. N Engl J Med. (2006) 355:788–98. 10.1056/NEJMoa055695 16928994

[3] Hiratzka LF Bakris GL Beckman JA Bersin RM Carr VF Casey DE Jr. 2010 ACCF/AHA/AATS/ACR/ASA/SCA/SCAI/SIR/STS/SVM guidelines for the diagnosis and management of patients with thoracic aortic disease: a report of the American College of Cardiology foundation/American Heart Association task force on practice guidelines, American association for thoracic surgery, American college of radiology, American stroke association, society of cardiovascular anesthesiologists, society for cardiovascular angiography and interventions, society of interventional radiology, society of thoracic surgeons, and society for vascular medicine. Circulation. (2010) 121:e266–369. 10.1161/CIR.0b013e3181d4739e 20233780

[4] Erbel R Aboyans V Boileau C Bossone E Bartolomeo RD Eggebrecht H 2014 ESC guidelines on the diagnosis and treatment of aortic diseases: document covering acute and chronic aortic diseases of the thoracic and abdominal aorta of the adult. The task force for the diagnosis and treatment of aortic diseases of the European Society of Cardiology (ESC). Eur Heart J. (2014) 35:2873–926. 10.1093/eurheartj/ehu281 25173340

[5] Isselbacher EM Preventza O Black JH 3rd Augoustides JG Beck AW Bolen MA 2022 ACC/AHA guideline for the diagnosis and management of aortic disease: a report of the American Heart Association/American College of Cardiology joint committee on clinical practice guidelines. Circulation. (2022) 146:e334–482. 10.1161/CIR.0000000000001106 36322642 PMC9876736

[6] Malaisrie SC Szeto WY Halas M Girardi LN Coselli JS Sundt TM 3rd 2021 The American association for thoracic surgery expert consensus document: surgical treatment of acute type A aortic dissection. J Thorac Cardiovasc Surg. (2021) 162:735–58.e2. 10.1016/j.jtcvs.2021.04.053 34112502

[7] Lee H Cho YH Sung K Kim WS Park KH Park PW Clinical outcomes of valve-sparing root replacement in acute type A aortic dissection. Scand Cardiovasc J. (2015) 49:331–6. 10.3109/14017431.2015.1072236 26166265

[8] Cho YH Sung K Kim WS Jeong DS Lee YT Park PW Malperfusion syndrome without organ failure is not a risk factor for surgical procedures for type A aortic dissection. Ann Thorac Surg. (2014) 98:59–64. 10.1016/j.athoracsur.2014.03.026 24820387

[9] Lee H Cho YH Sung K Kim WS Park KH Jeong DS Clinical outcomes of root reimplantation and bentall procedure: propensity score matching analysis. Ann Thorac Surg. (2018) 106:539–47. 10.1016/j.athoracsur.2018.02.057 29596818

[10] Min HK Sung K Yang JH Kim WS Jun TG Lee YT Can intraoperative motor-evoked potentials predict all the spinal cord ischemia during moderate hypothermic beating heart descending thoracic or thoraco-abdominal aortic surgery? J Card Surg. (2010) 25:542–7. 10.1111/j.1540-8191.2010.01080.x 20626514

[11] Aftab M Cikach FS Zhu Y Idrees JJ Rigelsky CM Kalahasti V Loeys-Dietz syndrome: intermediate-term outcomes of medically and surgically managed patients. J Thorac Cardiovasc Surg. (2019) 157:439–50.e5. 10.1016/j.jtcvs.2018.03.172 30669217

[12] Patel ND Crawford T Magruder JT Alejo DE Hibino N Black J Cardiovascular operations for Loeys-Dietz syndrome: intermediate-term results. J Thorac Cardiovasc Surg. (2017) 153:406–12. 10.1016/j.jtcvs.2016.10.088 27955909

[13] Seike Y Matsuda H Inoue Y Sasaki H Morisaki H Morisaki T The differences in surgical long-term outcomes between marfan syndrome and Loeys-Dietz syndrome. J Thorac Cardiovasc Surg. (2022) 164:16–25.e2. 10.1016/j.jtcvs.2020.07.089 32891455

[14] Williams JB McCann RL Hughes GC. Total aortic replacement in Loeys-Dietz syndrome. J Card Surg. (2011) 26:304–8. 10.1111/j.1540-8191.2011.01224.x 21443730 PMC3674881

[15] Schoenhoff FS Alejo DE Black JH Crawford TC Dietz HC Grimm JC Management of the aortic arch in patients with Loeys-Dietz syndrome. J Thorac Cardiovasc Surg. (2020) 160:1166–75. 10.1016/j.jtcvs.2019.07.130 31627951

