# Supplementary material for: Impact of early diagnosis on surgical outcomes in patients with Loeys-Dietz syndrome
Source: Front Cardiovasc Med. 2024 Aug 16;11:1429222. doi: 10.3389/fcvm.2024.1429222 (PMC11363540; doi:10.3389/fcvm.2024.1429222)
Supplement: Supplementary file 1 [file Table1.docx]

Supplementary Table S1. Baseline characteristics of patients who mismatched between diagnosis timing and emergency surgery.

| **Patient** | **Group** | **Sex** | **Age**  **(at first op.)** | **Diagnosis**  **(at first op.)** | **Op name**  **(at first op.)** | **Overall mortality** | **Freedom from reoperation** | **In-hospital morbidity and mortality** |
| --- | --- | --- | --- | --- | --- | --- | --- | --- |
| 1 | ED | Female | 64 | Acute type A dissection | Bentall op.  Ascending aorta and partial arch replacement | Survival | None | None |
| 2 | ED | Female | 34 | Acute type A dissection | Bentall op. | Survival | None | None |
| 3 | DD | Female | 18 | Annuloaortic ectasia | AV sparing (David op.) | Survival | None | None |
| 4 | DD | Female | 5 | Annuloaortic ectasia | AV sparing (David op.) | Survival | 18 months | None |
| 5 | DD | Male | 18 | Annuloaortic ectasia | AV sparing (David op.) | Survival | 128 months | None |
| 6 | DD | Male | 49 | Descending aorta aneurysm | Descending thoracic aorta replacement | Survival | 2 months | None |

Op, operation; ED, early diagnosis; DD, delayed diagnosis.
